# Changes in causes of pregnancy-related and maternal mortality in Zimbabwe 2007-08 to 2018-19: findings from two reproductive age mortality surveys

**DOI:** 10.1186/s12889-022-13321-7

**Published:** 2022-05-10

**Authors:** Reuben Musarandega, Solwayo Ngwenya, Grant Murewanhema, Rhoderick Machekano, Thulani Magwali, Lennarth Nystrom, Robert Pattinson, Stephen Munjanja, the Zimbabwe Maternal and Perinatal Mortality Study Group, Admire Chikutiro, Agnes Mahomva, Aveneni Mangombe, Bernard Madzima, Bothwell Guzha, Chipo Chimamise, Chipo Gwanzura, Davidzoyashe Makosa, Enesia Ziki, Esther Ngaru, Eunice Tahuringana, Gerald Madziyire, Grant Murewanhema, Gwendoline Chimhini, Jonathan Kasule, Julius Chirengwa, Lucia Gondongwe, Margaret Nyandoro, Maxwell Chirehwa, McMillan Parirenyatwa, Mercy Gaza, Michael Nyakura, Nhamo Gona, Reuben Musarandega, Ronald Mataya, Rumbidzai Makoni, Sarah Gunguwo, Thulani Magwali, Tsitsi Magure, Velda Mushangwe, Vongai Dondo, Winston Chirombe

**Affiliations:** 1 School of Health Systems and Public Health, University of Pretoria, Pretoria, South Africa; 2 Unit of Obstetrics and Gynaecology, Faculty of Medicine and Health Sciences, University of Zimbabwe, Harare, Zimbabwe; 3 Department of Obstetrics and Gynaecology, National University of Science and Technology, and Mpilo Central Hospital, Bulawayo, Zimbabwe; 4 Biostatistics and Epidemiology Department, Faculty of Medicine and Health Sciences, Stellenbosch University, Cape Town, South Africa; 5 Department of Epidemiology and Global Health, Umea University, Umea, Sweden; 6 Research Centre for Maternal, Fetal, Newborn & Child Health Care Strategies, University of Pretoria, Pretoria, South Africa

**Keywords:** Maternal death, Causes of death, CRVS, ICD-MM, ICD-10, Zimbabwe, Women of reproductive ages, Pregnancy-related deaths, Maternal mortality, International classification of diseases, Civil registration and vital statistics, Sustainable development goals, Zimbabwe

## Abstract

**Background:**

Reducing maternal mortality is a priority of Sustainable Development Goal 3.1 which requires frequent epidemiological analysis of trends and patterns of the causes of maternal deaths. We conducted two reproductive age mortality surveys to analyse the epidemiology of maternal mortality in Zimbabwe and analysed the changes in the causes of deaths between 2007-08 and 2018-19.

**Methods:**

We performed a before and after analysis of the causes of death among women of reproductive ages (WRAs) (12-49 years), and pregnant women from the two surveys implemented in 11 districts, selected using multi-stage cluster sampling from each province of Zimbabwe (*n*=10); an additional district selected from Harare. We calculated mortality incidence rates and incidence rate ratios per 10000 WRAs and pregnant women (with 95% confidence intervals), in international classification of disease groups, using negative binomial models, and compared them between the two surveys. We also calculated maternal mortality ratios, per 100 000 live births, for selected causes of pregnancy-related deaths.

**Results:**

We identified 6188 deaths among WRAs and 325 PRDs in 2007-08, and 1856 and 137 respectively in 2018-19. Mortality in the WRAs decreased by 82% in diseases of the respiratory system and 81% in certain infectious or parasitic diseases' groups, which include HIV/AIDS and malaria. Pregnancy-related deaths decreased by 84% in the indirect causes group and by 61% in the direct causes group, and HIV/AIDS-related deaths decreased by 91% in pregnant women. Direct causes of death still had a three-fold MMR than indirect causes (151 vs. 51 deaths per 100 000) in 2018-19.

**Conclusion:**

Zimbabwe experienced a decline in both direct and indirect causes of pregnancy-related deaths. Deaths from indirect causes declined mainly due to a reduction in HIV/AIDS-related and malaria mortality, while deaths from direct causes declined because of a reduction in obstetric haemorrhage and pregnancy-related infections. Ongoing interventions ought to improve the coverage and quality of maternal care in Zimbabwe, to further reduce deaths from direct causes.

**Supplementary Information:**

The online version contains supplementary material available at 10.1186/s12889-022-13321-7.

## Introduction

Reducing maternal mortality is a priority of Sustainable Development Goal (SDG) 3.1 and the target is to reduce the global maternal mortality ratio (MMR) to 70 maternal deaths per 100 000 live births by 2030 and to leave no country with an MMR greater than double the global average [1]. To achieve these targets,  systematic epidemiologic analysis of the levels, trends and causes of maternal deaths is required to periodically assess progress towards this goal [2–4]. Analysis of the MMR evaluates efforts to reduce the levels, while analysis of the causes of death identifies the aetiologies to target with ongoing interventions to reduce maternal mortality.

The World Health Organisation (WHO) developed the international classification of diseases (ICD), which groups diseases and causes of death into standard categories to aid their analysis. Currently, the 10^th^Edition of the ICD (ICD-10) is used to classify diseases and causes of death in the general population, including women of reproductive ages (WRAs) [5]. The 1^st^edition of the manual for classifying deaths during pregnancy, childbirth and the puerperium (ICD-MM) is currently used to classify pregnancy-related deaths [6, 7]. The ICD manuals enable standardised coding and description of diseases and causes of death globally.

Zimbabwe is among Sub-Saharan Africa (SSA) countries with high maternal mortality ratios (MMR), though the country's MMR has been gradually declining over the years. The Zimbabwe Demographic and Health Survey (ZDHS) reported an MMR of 960 in 2011 and 651 in 2015 [8, 9], the Multiple Indicator Cluster Surveys (MICS) in 2014 and 2019 estimated the MMR at 614 and 462 respectively [10, 11], while the United Nations Maternal Mortality Estimation Inter-agency Group (MMEIG) in 2017 estimated the MMR at 458 [12]. In 2017 the MMEIG estimated the MMR in SSA at 542, (917 in Nigeria, 524 in Tanzania, 401 in Ethiopia and 119 in South Africa). These high MMRs raise the need for studies that will identify the causes of maternal deaths to prioritise interventions regionally and globally.

### Methods

We conducted two reproductive age mortality surveys (RAMOS) to analyse the changes in Zimbabwe’s MMR and causes of death between 2007-08 and 2018-19. The study protocol has been published elsewhere [13]. This paper describes the important changes that occurred in the causes of reproductive age and maternal mortality in Zimbabwe during the review period. Another paper will report changes in the MMR. The RAMOS method was used because it provided data on the causes of death and applies to the use of secondary data sources.

### Interventions implemented

After the 2007-08 survey, Zimbabwe implemented several health interventions that could impact the country’s pregnancy-related and maternal deaths. Among these were HIV interventions, where the roll-out of antiretroviral therapy (ART) began in 2004 [14], and was scaled up with the number of health facilities providing ART increasing from 17% (282/1643) in December 2007 to 91% (166/1722) in December 2017 [15]. By 2019, 97% of adults (15-49 years), out of those who tested HIV-positive over time, were on ART [16]. For pregnant women, comprehensive HIV testing and treatment services were widely availed antenatally [17–20]. The "Option A" regimen was introduced in 2011 for the prevention of mother-to-child transmission (PMTCT) of HIV, in which pregnant and breastfeeding women in WHO clinical stage 3 or 4 or with a CD4 count $\le$350 cells/µl of blood were initiated on ART. In 2013, "Option B+" was introduced, where HIV-positive pregnant and breastfeeding women were initiated on life-long ART [17, 16]. As a result, more than 50% of HIV-positive pregnant women were already on ART at their first ANC visit in 2017 [21].

The government and its partners also implemented interventions to reduce the direct causes of maternal mortality. A maternal and neonatal health roadmap was developed to guide the prioritized interventions [22]. Doctors and nurses were trained throughout the country on basic and comprehensive emergency obstetric and newborn care (BEmONC and CEmONC) [23, 24]. BEmONC was introduced in primary healthcare clinics and CEmONC in district, provincial and central hospitals [23, 25, 26]. The training in emergency obstetric care supported the nurses, midwives and doctors to identify and manage signs and symptoms of the direct causes of maternal deaths such as obstetric haemorrhage, hypertensive disorders of pregnancy, obstetric trauma and others posing a danger to the mother and/or foetus, and hence institute timely life-saving interventions.

Guidelines for a maternal and perinatal death surveillance and response (MPDSR) system were developed [27]. Under these guidelines, the country started auditing maternal and perinatal deaths occurring in all health institutions [27]. The deaths were documented and reported on standard forms that health institutions submitted to the Ministry of Health and Child Care (MoHCC)’s head office where a national database was created to collect the data for all deaths reported [25]. MPDSR committees were established in hospitals and at district, provincial and national levels. The national MPDSR committee audited selected deaths and used the findings to develop and implement supervisory support and mentorship plans for the provinces and districts [27]. Maternity waiting homes which started in the 1980s were expanded to allow women to stay at maternity institutions from the third trimester until they delivered, so as to increase access to skilled delivery and emergency obstetric care when needed while reducing unsafe home deliveries [28–31]. The government removed user fees for maternity care in government-funded health institutions with the support of a multi-donor fund named the “health transition fund (HTF)” from 2012 to 2015 and the “health development fund (HDF)” from 2016 to 2020 [32–35]. The HTF and HDF supported the MPDSR activities, minor renovations in maternity institutions, procurement of basic commodities, medicines and equipment, and provided retention allowances for critical maternal health staff. Consequently, the use of maternity health care and institutional deliveries increased, as the ZDHS reported an increase in institutional deliveries from 65% in 2007-11 to 72% in 2012-16 [8, 9], while MICS reported 86% in 2015-19 [10]. Caesarean section deliveries also increased [36–38].

### Study design

A before and after analysis was performed using data from the two cross-sectional RAMOS conducted in 11 districts of the country to evaluate the impact on the causes of maternal mortality of the interventions implemented. The two surveys used multi-stage cluster sampling, first stratifying the population into provinces (n=10), and selecting one district from each province using simple random sampling. An additional district was selected in Harare, because of the presence of two referral hospitals in the capital city province, to which complicated maternal cases were referred from other provinces. The surveys collected data for births and deaths among WRAs, including maternal deaths. The surveys were designed to produce representative samples of births (45000 and 46000 respectively), needed to calculate the MMR. The study protocol describes in detail the sampling procedures and sample size calculations [13].

### Study setting

**Fig. 1:**
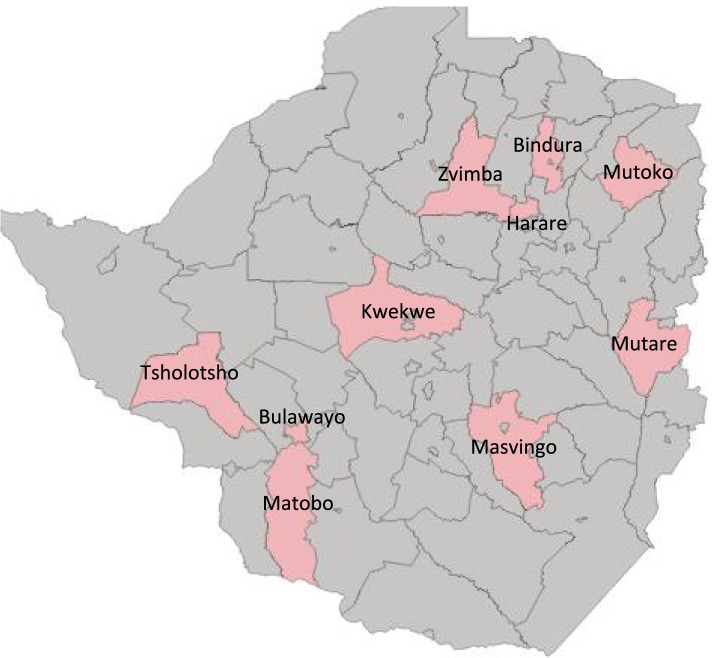
Map of Zimbabwe maternal and perinatal mortality study sites

The 11 study districts (provinces) were: Mutare (Manicaland), Mutoko (Mashonaland East), Bindura (Mashonaland Central), Zvimba (Mashonaland West), Chivi (Masvingo), Kwekwe (Midlands), Tsholotsho (Matabeleland North), Matobo (Matabeleland South), Nkulumane (Bulawayo), and South-Eastern and Western (Harare). Mutare, Bindura and Kwekwe are partly urban districts. Nkulumane, Harare South Eastern, and Harare Western are urban districts (See Figure 1).

### Data collected

Data were collected for deaths among WRAs on location (province, district and place of residence – rural/urban), age (completed years), pregnancy status (pregnant or not), and causes of death (as stated on medical and death certificates). For pregnancy-related deaths, we collected data on complications suffered and birth outcomes, institutions where the patient was referred to (level of the facility – district, provincial or tertiary hospital, and reasons for referral), causes of death (as above) and place of death (home or institutional).

### Data collection procedures

Secondary data were collected from civil registration and vital statistics (CRVS) records at the government’s Registrar General’s (RG’s) offices and health facility records. The health records included patient registers and charts at the following sites: maternity units, theatres, high dependency and intensive care units, gynaecological, medical and surgical wards, mortuaries, hospital police posts, and casualty departments. In addition, village health workers and village heads recorded home deaths in community registers in 2007-08, and trained research nurse-midwives interviewed the deceased women's close relative present at the time of death (husband, mother, sister or other) using verbal autopsy forms adapted from the WHO [39]. In 2018-19, additional deaths were identified in maternal death notification forms from the Ministry of Health and Child Care's district, provincial and national reproductive health offices.

### Civil registration and vital statistics (CRVS) policy and procedures

The CRVS and health system data are regulated by government legislation in Zimbabwe. The law enforces registration and issuance of certificates for all deaths [40], and requires health institutions and relatives or village heads of persons who die at home to notify the death at the RG's office to create a death record and issue a death certificate. Medical officers or nurses who attend a death in a health institution complete and sign a medical death certificate. Family members report home deaths to the police, which takes the bodies to hospitals, where medical officers perform post-mortems and issue medical death certificates with causes of death. Health institutions submit the medical death certificates to RG's district offices, where death records are created. The RG's officers file the death records by year and date and store them in secure record rooms.

### Data collection period

Data collection for the first survey was conducted prospectively between 1st May 2007 and 15th June 2008 and repeated retrospectively between 1st May and 31st July 2020. For the second survey covering the period 1 May 2018 to 15 June 2019, data collection was done retrospectively between 1st May and 31^st^ July 2020 and repeated between 3rd May and 20th July 2021.

### Definitions

WRAs were women aged 15 to 49 years. Pregnancy-related deaths were deaths during pregnancy or within 42-days of termination of pregnancy or delivery, irrespective of the cause of the termination of pregnancy and death [12]. Maternal deaths were deaths of women during pregnancy or within 42-days of termination of pregnancy or delivery, irrespective of the duration and site of the pregnancy, from any cause related to or aggravated by the pregnancy or its management, but not from accidental or incidental causes [6, 12].

### Eligibility criteria and participant selection

Deaths among WRAs resident in the study districts who died from any cause, in health institutions or home, with their death records filed in the district RG's office were eligible for the study, including those who died in referral hospitals in other districts. Similarly, pregnancy-related deaths of district residents, which occurred in the local or referral hospitals, at home or in transit, were eligible for the study.

### Data analysis

A before-and-after analysis of the changes in causes of reproductive age and maternal mortality was performed. Mortality incidence rates (IR) and incidence rate ratios (IRR) (per 10 000 WRA) with 95% confidence intervals (CIs) were calculated in ICD-10 groups, and IR and IRR (per 10 000 deliveries) were calculated with 95% CIs for pregnancy-related deaths in ICD-MM groups and specific causes. Cause-specific MMRs were also calculated with 95% CIs for the leading causes of pregnancy-related deaths. We used negative binomial models in the analysis, which treated the two surveys as cohorts because of the before-and-after design and used mortality incidence rate ratios to quantify changes in mortality levels of each cause of death between the two surveys. We used STATA version 17.0’s immediate commands [41], to perform the analysis because the denominators of the ratios were aggregate data. The 95% CIs for IRRs that contained “1” were considered not statistically significant. Percentages of maternal deaths in ICD-MM groups (out of total maternal deaths) were calculated. The leading causes of maternal deaths were ranked according to the calculated percentage and compared with Southern Africa (SA), and Sub-Saharan Africa (SSA) estimates using data from a recent systematic review [42].

### Strengths of the study

The study has the strength that it collected data to estimate changes in the MMR together with changes in the causes of death, to provide comprehensive recommendations for reducing the mortality. The two surveys triangulated data from various sources (civil registration records, patient health records, and maternal death notification forms and databases), which enhanced the identification of the deaths and their causes. The study used ICD-10 and ICD-MM manuals to classify and code the causes of death and the classification was done by trained obstetricians using the ICD manuals. This minimized misclassification of the deaths and their causes, and the ICD coding makes the findings interpretable within global trends.

## Results

### Deaths among women of reproductive ages (WRAs)

We identified 6 188 WRAs in 2007-08 and 1 856 in 2018-19 (Table 1). Majority of the deaths of WRAs were in 12 cause groups in both years; 96% in 2007-08 and 89% in 2018-19. The ICD-10 group “certain infectious or parasitic diseases” constituted 60% of all deaths in 2007-08 and 44% in 2018-19. HIV/AIDS constituted 96% of deaths in this group (3568/3728 and 790/823 respectively) and was the leading cause of death in both study periods; causing 58% (3568/6188) of deaths in 2007-08 and 43% (790/1856) of deaths in 2018-19.

**Table 1:** Causes of death among women of reproductive age (15-49 years) in Zimbabwe, 2007-08 and 2018-19

**Cause of death (ICD-10 group)**	**2007-08**		**2018-19**		**IRR (95% CI)**
	**Number of deaths**	**IR/10000**	**Number of deaths**	**IR/10000**	
Certain infectious or parasitic diseases*	3728	62.0	823	11.7	0.19 (0.18-0.21)
HIV/AIDS*	3568	59.4	790	11.2	0.19 (0.17-0.20)
Malaria*	159	2.6	32	0.45	0.17 (0.11-0.25)
Neoplasms	100	1.7	106	1.5	0.90 (0.68-1.2)
Diseases of the blood or blood-forming organisms	39	0.65	27	0.38	0.59 (0.35-1.0)
Endocrine, nutritional, or metabolic diseases	38	0.63	32	0.45	0.72 (0.43-1.2)
Diseases of the nervous system	296	4.9	103	1.5	0.30 (0.23-0.37)
Diseases of the circulatory system	252	4.2	122	1.7	0.41 (0.33-0.51)
Diseases of the respiratory system	480	8.0	103	1.4	0.18 (0.15-0.23)
Diseases of the digestive system	599	10.0	148	2.1	0.21 (0.17-0.25)
Diseases of the musculoskeletal system and connective tissue	25	0.42	8	0.11	0.27 (0.11-0.62)
Diseases of the genitourinary system	23	0.38	36	0.51	1.3 (0.77-2.4)
Pregnancy, childbirth, and the puerperium	325	5.4	137	1.9	0.36 (0.29-0.44)
Injury, poisoning, and certain other consequences of external causes	118	2.0	109	1.5	0.79 (0.60-1.0)
Other groups**	8	-	4	-	-
Insufficient information	240	-	177	-	-
Unknown	21	-	20	-	-
**Total deaths**	6188	103.1	1856	26.4	0.26 (0.24-0.27)
**Population**	600344	-	704176	-	-

^*The group includes cases that are in group 18 but were due to infections in group 1.^

^**For 2007: Diseases of the immune system - 1; mental, behavioural or neurodevelopment -2; diseases of the skin -3, conditions related to sexual health -1, developmental anomalies -1; For 2018: Diseases of the immune system -1; diseases of the ear or mastoid process -1; diseases of the skin -8.^

Mortality declined significantly in six groups: certain infectious or parasitic diseases (81%), diseases of the respiratory system (82%), diseases of the nervous system (70%), diseases of the circulatory system (69%), diseases of the digestive system (79%) and pregnancy and childbirth (64%).

### Pregnancy-related deaths

We identified 325 pregnancy-related deaths in 2007-08 and 137 in 2018-19 (Table 2). Direct causes accounted for 55% (180/325) of the deaths in 2007-08 and 75% (103/137) in 2018-19. Deaths due to direct causes decreased by 61%. Within this category, deaths more than halved in three groups– hypertensive disease in pregnancy (50%), obstetric haemorrhage (64%), pregnancy-related infections (86%), and the largest decline occurred in puerperal sepsis (91%).

**Table 2:** Causes of pregnancy-related deaths in Zimbabwe, 2007-08 and 2018-19; Incidence rate (IR) and Incidence rate ratio (IRR) per 10 000 and 95% confidence intervals (CI)

Cause of death (ICD10-MM Group and specific causes)^*^	**2007-2008**		**2018-2019**		**IRR (95% CI)**
	**Number of deaths**	**IR/10000**	**Number of deaths**	**IR/10000**	
**Direct causes**	**180 (55%)**	**39.5**	**103 (75%)**	**15.3**	**0.39 (0.30-0.50)**
**1 Pregnancies with abortive outcome**	**30**	**6.6**	**24**	**3.6**	**0.54 (0.30-0.96)**
Unsafe/septic /complications of abortion	19	4.2	12	1.8	0.43 (0.19-0.93)
Ectopic pregnancy/ruptured ectopic pregnancy	7	1.5	7	1.0	0.68 (0.20-2.3)
Peri-abortal haemorrhage	4	0.88	5	0.74	0.85 (0.18-4.3)
**2 Hypertensive disorders in pregnancy**	**34**	**7.5**	**25**	**3.7**	**0.50 (0.29-0.86)**
Severe/Pre-eclampsia/Eclampsia	34	7.5	22	3.3	0.44 (0.24-0.77)
Hypertensive disease in pregnancy	0	0.0	3	0.44	-
**3 Obstetric haemorrhage**	**64**	**14.0**	**34**	**5.1**	**0.36 (0.23-0.55)**
Unspecified obstetric haemorrhage	26	5.7	17	2.5	0.44 (0.23-0.85)
Postpartum haemorrhage/PPH	26	5.7	11	1.6	0.29 (0.13-0.60)
Antepartum haemorrhage/APH	4	0.88	2	0.30	0.34 (0.03-2.4)
Ruptured uterus/Uterine rupture	8	1.8	4	0.60	0.34 (0.07-1.27)
**4 Pregnancy-related infections**	**33**	**7.2**	**7**	**1.0**	**0.14 (0.05-0.33)**
Puerperal sepsis	32	7.0	4	0.60	0.08 (0.02-0.24)
Chorioamnionitis/Septicaemia	1	0.22	3	0.45	2.0 (0.16-106.8)
**5 Other obstetric complications**	**14**	**3.1**	**12**	**1.8**	**0.58 (0.25-1.4)**
Obstructed/prolonged labour	7	1.5	1	0.14	0.10 (0.0-0.75)
Obstetric/pulmonary embolism	3	0.66	2	0.30	0.45 (0.04-3.9)
Cardiomyopathy/Postpartum cardiomyopathy	3	0.66	8	1.2	1.8 (0.43-10.6)
Hyperemesis gravidarum	1	0.22	1	0.14	0.68 (0.01-53)
**6 Unanticipated complications of management**	**5**	**1.1**	**1**	**0.14**	**0.14 (0.0-1.2)**
Anaesthetic complications/High spinal anaesthesia	5	1.1	0	0.0	0.0 (0.0-0.74)
High spinal	0	0.0	1	0.14	-
**Indirect causes**	**145 (45%)**	**31.8**	**34 (26%)**	**5.2**	**0.16 (0.11-0.24)**
**7 Non-obstetric complications**	**113**	**22.6**	**29**	**4.2**	**0.18 (0.12-0.28)**
HIV/AIDS	72	15.8	10	1.5	0.09 (0.04-0.18)
Malaria	23	5.0	2	0.30	0.06 (0.01-0.24)
Tuberculosis	8	1.8	2	0.30	0.17 (0.02-0.85)
Cardiac disease	5	1.1	3	0.45	0.41 (0.06-2.1)
Other indirect causes	5	1.1	12	1.8	1.6 (0.53-5.9)
**8 Unknown/undetermined causes**	**19**	**4.2**	**3**	**0.45**	**0.11 (0.02-0.36)**
Unknown/unspecified causes	19	4.2	3	0.45	0.11 (0.02-0.36**)**
**9 Coincidental causes**	**13**	**2.9**	**2**	**0.30**	**0.10 (0.01-0.46)**
Assault, Poisoning, Suicide, RTA	13	2.9	2	0.30	0.10 (0.01-0.46)
**Total deaths**	325	71.3	137	20.4	0.29 (0.23-0.35)
**Total deliveries**	45579	-	67225	-	-

Deaths due to indirect causes decreased by 84%, with deaths due to non-obstetric complications decreasing by 82%. The largest declines occurred in HIV/AIDS (91%), and malaria (94%). HIV/AIDS dropped from being the top cause of death in 2007-08 (16 deaths per 10 000) to fourth cause (2 deaths per 10 000) in 2018-19, behind the direct causes of eclampsia (3 deaths per 10 000), abortion-related complications (4 deaths per 10 000) and postpartum haemorrhage (3 deaths per 10 000).

Direct causes of death had a three-fold MMR (151 maternal deaths per 100 000) than indirect causes (51 maternal deaths per 100 000) in 2018-19. Obstetric haemorrhage alone had the same MMR as all indirect causes of death (Table 3).

**Table 3:** Cause-specific maternal mortality ratio (MMR) for the selected cause of death groups in Zimbabwe, 2018-19

**Cause of death group**	**Maternal deaths**	**MMR**^**1**^** (95% CI)**
**Direct causes**	**103**	**153 (125 – 186)**
Obstetric haemorrhage	34	51 (35 – 71)
Pregnancies with abortive outcome	24	38 (23 – 53)
Hypertensive disorders in pregnancy	25	37 (24 – 55)
Other obstetric complications	12	18 (9 – 31)
Pregnancy-related infections	7	10 (4 – 21)
**Indirect causes**	**34**	**51 (35 – 71)**
Non-obstetric complications	29	43 (29 – 62)
HIV/AIDS	10	15 (7 – 27)

^1^ MMR = (number of maternal deaths ÷ number of live births) x 100 000. The number of live births was 67225 in 2018-19.

## Discussion

Analysing the causes of pregnancy-related and reproductive age mortality together provides important insights into the reasons for the decline. Sometimes declines in pregnancy-related mortality are associated with declines in WRAs because the interventions for non-obstetric diseases and causes of death in the general population benefits pregnant women as well [2–4].

This study found significant declines in deaths due to different causes in WRAs and pregnant women in Zimbabwe from 2007-08 to 2018-19. Mortality among WRAs significantly declined in six out of twelve ICD-10 groups; mainly among groups associated with HIV/AIDS, such as certain infectious and parasitic diseases, respiratory system, and digestive system diseases. HIV and malaria are the main infectious and parasitic diseases in Zimbabwe [5]. Respiratory diseases such as pulmonary tuberculosis and bacterial pneumonia and digestive system diseases such as acute gastroenteritis are AIDS-related in high HIV burden countries [43, 44]. Zimbabwe has achieved significant reductions in HIV-related [45–47] and malaria deaths [48]. Improvement in the provision of antiretroviral therapy (ART) has reduced HIV-related mortality significantly. On the contrary, mortality among WRAs from non-infectious diseases remained stable, consistent with observed trends of non-communicable diseases (NCDs) in Africa [49, 50].

Pregnancy-related deaths from indirect causes decreased by more than four-fifths (84%), of which HIV/AIDS was the leading cause in 2007-08, before the widespread availability of antiretroviral therapy (ART) [14]. A that time, significant proportions of pregnant women went through their antenatal period not knowing their HIV status [46, 47]. By 2018-19, the situation had changed significantly. HIV/AIDS dropped to the fourth cause of death, after the direct causes of abortion, eclampsia, and postpartum haemorrhage.

In 2018-19, the leading causes of maternal deaths in Zimbabwe (obstetric haemorrhage, hypertensive disease in pregnancy and non-obstetric causes) were the same as in the Southern Africa (SA) and Sub-Saharan Africa (SSA) regions (Table 4) [42]. Abortion-related deaths were higher in Zimbabwe than SA and SSA (18% vs. 8% and 7%), despite the known challenges of identifying them because of prohibitive legislation and religious objections [51–53]. Unanticipated complications of management deaths were possibly poorly reported because medical staff fear blame and litigation [54, 55].

**Table 4:** Comparison of the leading causes of pregnancy-related deaths in ICD-MM groups in Zimbabwe, Southern Africa and Sub-Saharan Africa, 2018-2019

**ICD-MM group and cause of death**	**Zimbabwe, 2018-2019**			**Southern Africa (SA), 2018-2019**			**Sub-Saharan Africa (SSA), 2018-2019**	
	Percentage (95% CI)	Rank	Percentage (95% CI)		Rank	Percentage (95% CI)		Rank
O3: Obstetric haemorrhage	25% (18%-33%)	1	25% (24%-27%)		1	29% (27%-31%)		1
O7: Non-obstetric complications	21% (15%-29%)	2	23% (22%-24%)		2	19% (16%-21%)		3
O2: Hypertensive disorders in pregnancy	18% (12%-26%)	3.5	18% (17%-19%)		3	22% (20%-24%)		2
O1: Pregnancies with abortive outcome	18% (12%-26%)	3.5	7.5% (6.7%-8.4%)		6	7.2% (5.3%-9.1%)		5
O5: Other obstetric complications	7.3% (3.6%-13%)	5	3.6% (3.1%-4.3%)		7	5.0% (3.1%-7.0%)		6
O4: Pregnancy-related infections	4.4% (1.6%-9.3%)	6	8.8% (8%-10%)		5	12% (10%-13%)		4
O8: Unknown/undetermined causes	2.1% (0.5%-6.3%)	7	3.5% (2.9%-4.1%)		8	2.5% (0.0%-4.9%)		8
O9: Coincidental causes	1.0% (0.0%-5.0%)	8	0.0%		9	0.0%		9
O6: Unanticipated complications of management	0.7% (0.0%-4.0%)	9	10% (10%-11%)		4	4.0% (1.6%-6.3%)		7
**Total deaths**	137		3736			11 431		

Zimbabwe halved deaths due to direct causes of maternal deaths (hypertension, haemorrhage, pregnancy-related infections) and the indirect causes of HIV/AIDS and malaria. Interventions implemented at various levels of the health system: policy development (roadmap), training (EmONC), providing access (maternity waiting homes, removal of user fees), monitoring and evaluation (MPDSR) achieved this impact. The achievements demonstrated how concerted multipronged interventions can reduce maternal mortality.

Notwithstanding, direct causes continued to cause maternal deaths more than indirect, with a three-fold cause-specific MMR. Direct causes are continuing to contribute significantly to maternal deaths because of the ever-present threat of brain drain where skilled healthcare workers migrate to better-remunerating countries [56]. Zimbabwe has also seen intermittent supplies of life-saving resources, such as blood products for obstetric haemorrhage, oxytocin – the standard uterotonic drug for managing the active third stage of labour and treating patients with post-partum haemorrhage. Similarly, stock ruptures of magnesium sulphate for eclampsia and anti-hypertensive medicines have been reported from time to time [57, 58]. Sustained investment in the supply of these life-saving drugs and resources is required to reduce deaths from direct causes.

Addressing obstetric haemorrhage would reduce deaths from direct causes by a third while addressing obstetric haemorrhage, abortion and hypertensive diseases would reduce deaths due to direct-causes by four-fifths. Thus, improving the coverage and quality of maternity care targeting these three causes remains a priority. Despite the presence of maternal waiting shelters in health facilities, women continue to experience significant first, second and third delays from various controllable factors, such as long distances to the nearest health facility and significant delays in getting transport to referral centres, coupled with human resource and commodity challenges at the referral centres. Efforts to reduce unskilled deliveries at home and ill-equipped primary-care facilities, improve emergency transport and increase access to the right care should continue [59–63]. Diagnosis and treatment of hypertensive diseases in pregnancy must also be prioritised as NCDs increase in SSA [49, 50, 64, 65].

## Study limitations

The limitation of this study is the possible under-representation of community deaths in 2018-19 data. The 2007-08 survey collected home deaths in the community, while the 2018-19 survey only included community deaths that were recorded in maternal death notification and CRVS records. Missing information for some deaths was addressed by triangulating the deaths across data sources. Deaths identified in CRVS records with incomplete information and not found in health records were classified as deaths of unknown causes, but these were few. Despite these limitations, the study identified sufficient deaths that were thoroughly reviewed by obstetricians to produce these findings.

## Conclusion

Mortality due to HIV and malaria has declined significantly in Zimbabwe in WRAs and pregnant women, though the two remain important causes of death. Zimbabwe has also significantly reduced pregnancy-related deaths from direct causes (pregnancy-related infections, obstetric haemorrhage and hypertensive disease in pregnancy) through concerted multipronged interventions. Sustained investment into the health system focusing on improving coverage and quality of antenatal care and access to emergency obstetric care will further reduce deaths from direct causes (haemorrhage, eclampsia and abortion). Efforts to contain the indirect causes of HIV and malaria should continue whilst increasing efforts to manage NCDs in pregnancy. The RAMOS should be repeated in the same districts before 2030, to assess progress towards the SDG target.

## Supplementary Information

**Additional file 1.** 

**Additional file 2.**

## Data Availability

The dataset supporting the conclusions of this article will be available to the public via this link: https://drive.google.com/drive/folders/1hOH_V9AjVXG6Sefdhe7QWOOnZacuM-sL

## Abbreviations

ART: Antiretroviral therapy
BD: Birth and death
CI: Confidence interval
CRVS: Civil registration and vital statistics
DHS: Demographic and health survey
DHIS: District health information system
ICD: International classification of diseases
MPDSR: Maternal and perinatal death surveillance and response
MDG: Millennium development goal
MICS: Multiple indicator cluster survey
MMR: Maternal mortality ratio
PRD: Pregnancy-related death
RAMOS: Reproductive age mortality survey
RG: Registrar general
SSA: Sub-Saharan Africa
SDG: Sustainable development goal
TFR: Total fertility rate
VA: Verbal autopsy
WRA: Women of reproductive age
