# Supplementary material for: Changes in causes of pregnancy-related and maternal mortality in Zimbabwe 2007-08 to 2018-19: findings from two reproductive age mortality surveys
Source: BMC Public Health. 2022 May 10;22:923. doi: 10.1186/s12889-022-13321-7 (PMC9087911; doi:10.1186/s12889-022-13321-7)
Supplement: Supplementary file 1 — Additional file 1. [file 12889_2022_13321_MOESM1_ESM.docx]

**Zimbabwe Maternal and Perinatal Mortality Study (ZMPMS) Members who contributed to the two surveys but not the authorship of the paper:**

| **ZMPMS 2007-08 Group**  Gwendoline Kandawasvika  Davidzoyashe Makosa  Maxwell Chirehwa  Eunice Tahuringana  Margaret Nyandoro  Aveneni Mangombe  Esther Ngaru  Tsitsi Magure  Nhamo Gona  Vongai Dondo  Ronald Mataya  Bothwell Guzha  Jonathan Kasule  Taurai Gunguwo  Sarah Manyame  Julius Chirengwa  Velda Mushangwe | **ZMPMS 2018-19 Group**  Michael Nyakura  Gerald Madziyire  Gwendoline Chimhini  Sunhurai Mukwambo  McMillan Parirenyatwa  Agnes Mahomva  Bernard Madzima  Davidzoyashe Makosa  Lucia Gondongwe  Chipo Chimamise  Winston Chirombe  Enesia Ziki  Mercy Gaza  Chipo Gwanzura  Admire Chikutiro  Rumbidzai Makoni |
| --- | --- |
