# Supplementary material for: Changes in causes of pregnancy-related and maternal mortality in Zimbabwe 2007-08 to 2018-19: findings from two reproductive age mortality surveys
Source: BMC Public Health. 2022 May 10;22:923. doi: 10.1186/s12889-022-13321-7 (PMC9087911; doi:10.1186/s12889-022-13321-7)
Supplement: Supplementary file 2 — Additional file 2. [file 12889_2022_13321_MOESM2_ESM.docx]

**Table 5: Change in cause-specific maternal mortality ratio (MMR) in eleven districts in Zimbabwe 2007-08 and 2018-19, incidence rate ratio (IRR) and 95% confidence intervals (CI)**

| **District** | **Number of births** | | **Deaths from direct causes** | | | **Deaths from indirect causes**  **(Non-obstetric complications)** | | | **Deaths from obstetric haemorrhage** | | | **Deaths from pregnancies with abortive outcome** | | | **Deaths from hypertensive disorders in pregnancy** | | |
| --- | --- | --- | --- | --- | --- | --- | --- | --- | --- | --- | --- | --- | --- | --- | --- | --- | --- |
|  | **2007-08** | **2018-19** | **2007-08** | **2018-19** | **IRR (95% CI)** | **2007-08** | **2018-19** | **IRR (95% CI)** | **2007-08** | **2018-19** | **IRR (95% CI)** | **2007-08** | **2018-19** | **IRR (95% CI)** | **2007-08** | **2018-19** | **IRR (95% CI)** |
| Nkulumane^1^ | 4002 | 5363 | 35 | 15 | 0.32 (0.16-0.60) | 14 | 4 | 0.21 (0.05-0.68) | 14 | 4 | 0.21 (0.05-0.68) | 5 | 3 | **0.45 (0.07-2.3)** | 7 | 4 | **0.43 (0.09-1.7)** |
| Harare SE^1^ | 1911 | 2240 | 9 | 4 | **0.38 (0.09-1.4)** | 3 | 0 | - | 1 | 3 | **2.5 (0.21-134)** | 3 | 0 | - | 2 | 0 | - |
| Harare W^1^ | 4958 | 7081 | 24 | 13 | 0.38 (0.18-0.78) | 7 | 6 | **0.60 (0.17-2.1)** | 4 | 2 | **0.35 (0.03-2.4)** | 4 | 4 | **0.70 (0.13-3.8)** | 12 | 3 | 0.18 (0.03-0.65) |
| Mutare | 7975 | 13200 | 39 | 20 | 0.31 (0.17-0.54) | 17 | 8 | 0.28 (0.11-0.70) | 13 | 6 | 0.28 (0.09-0.79) | 5 | 7 | **0.85 (0.23-3.4)** | 5 | 5 | **0.6 (0.14-2.6)** |
| Bindura | 3016 | 5982 | 13 | 6 | 0.23 (0.07-0.66) | 11 | 2 | 0.09 (0.01-0.42) | 8 | 0 | - | 2 | 3 | **0.76 (0.09-9.1)** | 2 | 2 | **0.5 (0.04-6.9)** |
| Mutoko | 3035 | 5348 | 11 | 10 | **0.52 (0.20-1.3)** | 12 | 2 | 0.09 (0.01-0.42) | 3 | 5 | **0.95 (0.18-6.1)** | 2 | 3 | **0.85 (0.1-10)** | 0 | 2 | - |
| Zvimba | 5863 | 8316 | 15 | 10 | **0.47 (0.19-1.1)** | 7 | 3 | **0.30 (0.05-1.3)** | 5 | 5 | **0.71 (0.16-3.1)** | 7 | 1 | 0.10 (0.02-0.78) | 1 | 3 | **2.1 (0.17-111)** |
| Chivi | 3834 | 4863 | 5 | 5 | **0.79 (0.18-3.4)** | 15 | 1 | 0.05 (0.0-0.34) | 3 | 2 | **0.53 (0.04-4.6)** | 0 | 1 | - | 1 | 1 | **0.79 (0.01-62)** |
| Tsholotsho | 2697 | 3221 | 4 | 3 | **0.63 (0.09-3.7)** | 8 | 0 | - | 2 | 1 | **0.42 (0.01-8.0)** | 0 | 0 | - | 0 | 2 | - |
| Matobo | 1961 | 2569 | 3 | 4 | **1.0 (0.17-6.9)** | 3 | 0 | - | 1 | 1 | **0.76 (0.01-60)** | 1 | 0 | - | 0 | 2 | - |
| Kwekwe | 6327 | 9042 | 23 | 13 | 0.40 (0.18-0.81) | 7 | 3 | **0.30 (0.05-1.3)** | 12 | 5 | 0.29 (0.08-0.89) | 2 | 3 | **1.0 (0.12-13)** | 4 | 1 | **0.17 (0.004-1.8)** |
| **Total** | **45,579** | **67225** | **181** | **103** | **0.39 (0.30-0.49)** | **104** | **29** | **0.19 (0.12-0.29)** | **66** | **34** | **0.35 (0.22-0.54)** | **31** | **25** | **0.55 (0.31-0.96)** | **34** | **25** | **0.92 (0.53-1.6)** |
| ^1^ Nkulumane district in Bulawayo, and South Eastern and Western districts in Harare are entirely urban districts;  Bold CIs are statistically not significant as they intervals contain 1;  CIs marked (-) were null because the number of deaths in one study year is 0. | | | | | | | | | | | | | | | | | |
